# Not All Fun and Games: A Case Report of Contact Dermatitis Related to Slime and Play-Doh

**DOI:** 10.7759/cureus.10272

**Published:** 2020-09-06

**Authors:** Brett C Brazen, Brooke Wehausen, Andleeb A Usmani

**Affiliations:** 1 Dermatology, Nova Southeastern University - Kiran C. Patel College of Osteopathic Medicine, Palm Harbor, USA; 2 Dermatology, Broward Health Medical Center, Fort Lauderdale, USA; 3 Dermatology, Nicklaus Children’s Hospital, Miami, USA

**Keywords:** dermatology, contact dermatitis, allergic dermatoses, genodermatoses, irritant contact dermatitis, medical dermatology

## Abstract

Slime and Play-Doh have been gaining popularity among adolescents and preteens. Both are gooey, viscoelastic substances that can be morphed and molded into virtually anything imaginable. Slime can be made from several household products including school glue and borax, which have both been linked to cases of irritant and allergic contact dermatitis, conditions that historically involve activation of many immune-regulatory cells such as Langerhans' cells and T cells. We review the rising incidence of contact dermatitis related to Slime and Play-Doh, pathogenesis, and hallmark findings, along with several treatment options to help clinicians expediently diagnose and treat the condition.

## Introduction

Allergic contact dermatitis (ACD) is an oft-seen cutaneous reaction, especially in young children. The most common causes are nickel, fragrances, and preservatives [1]. ACD occurs in two phases: the sensitization phase and the elicitation phase. In the sensitization phase, specialized dendritic cells known as Langerhans' cells (LCs) react along with adjacent keratinocytes to allergens or haptens (large proteins that require a carrier molecule to elicit a response). These LCs carry the allergens to subcutaneous lymphoid tissues where they present haptens via major histocompatibility complex (MHC) complexes on the cell surface to naive allergen-specific T cells, resulting in the production of memory T-cells, a process known as priming. The elicitation phase of ACD produces the systemic manifestations we typically see clinically. This process is due to the activation of the primed memory T-cells, typically CD8^+^ T_H_1 cells, upon re-exposure to the offending hapten. Activation of the CD8^+^ T-cells induces apoptosis of local keratinocytes, activation of the Fas/FasL pathway, and release of inflammatory cytokines interferon-gamma (IFN-y), and tumor necrosis factor-alpha (TNF-a), thus causing the cutaneous and systemic manifestations of ACD [1]. Our case is unique in that it features a case of contact dermatitis sparked by unusual contaminants, Play-Doh and slime, two staple childhood toys that are often concocted from a variety of ingredients. Our case report explores the pathophysiology of ACD, its similarities, and differences from irritant contact dermatitis (ICD), and discusses common treatment modalities. 

## Case presentation

**Figure 1:**
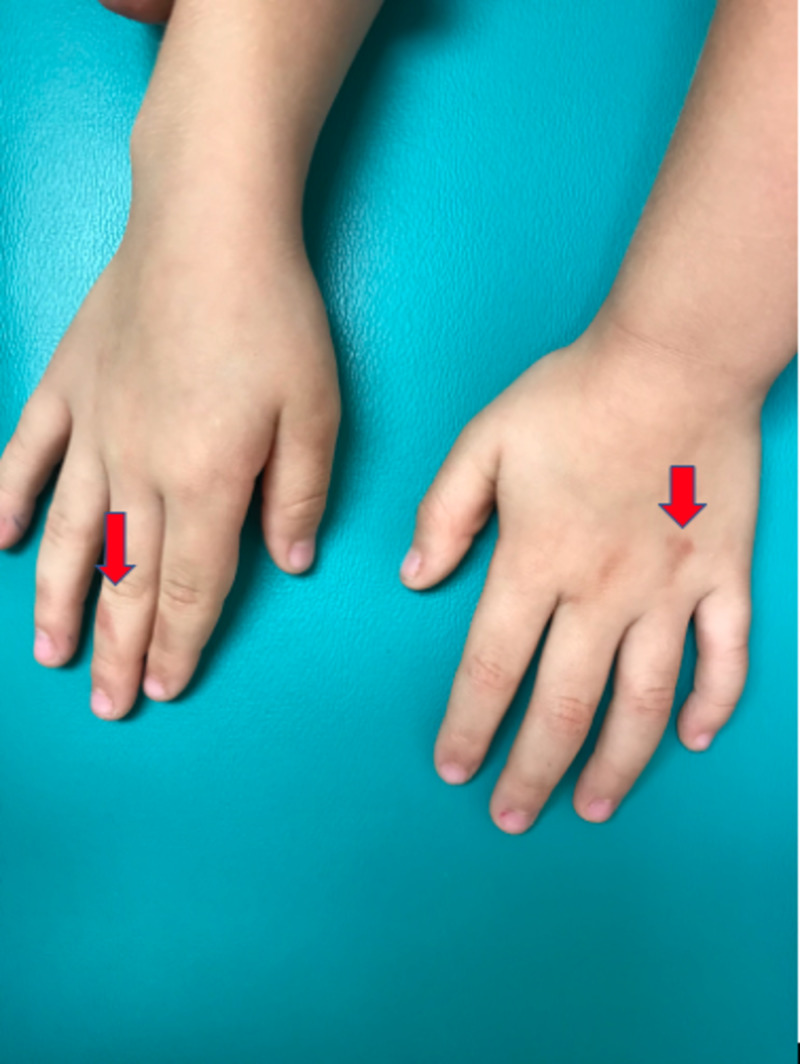
Hyperpigmented macules on the dorsum of the left hand and second to fourth interphalangeal joints of the right hand (superior view)

**Figure 2:**
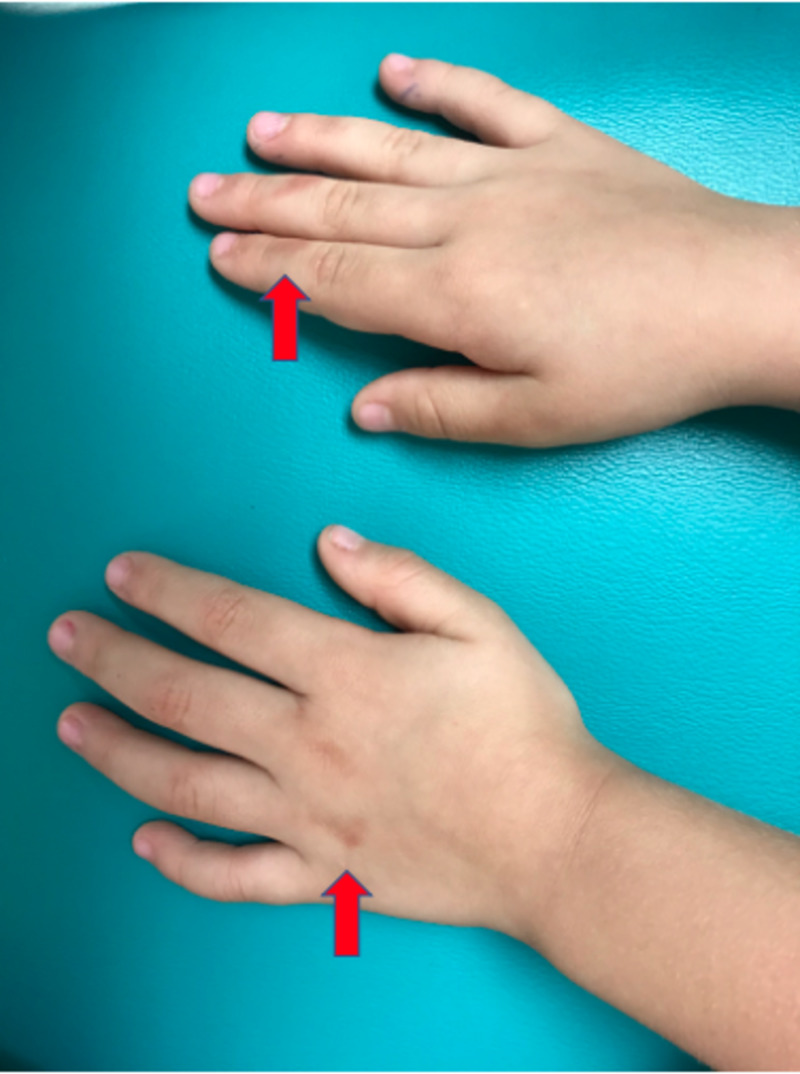
Hyperpigmented macules on the dorsum of the left hand and second to fourth interphalangeal joints of the right hand (superolateral view)

**Figure 3:**
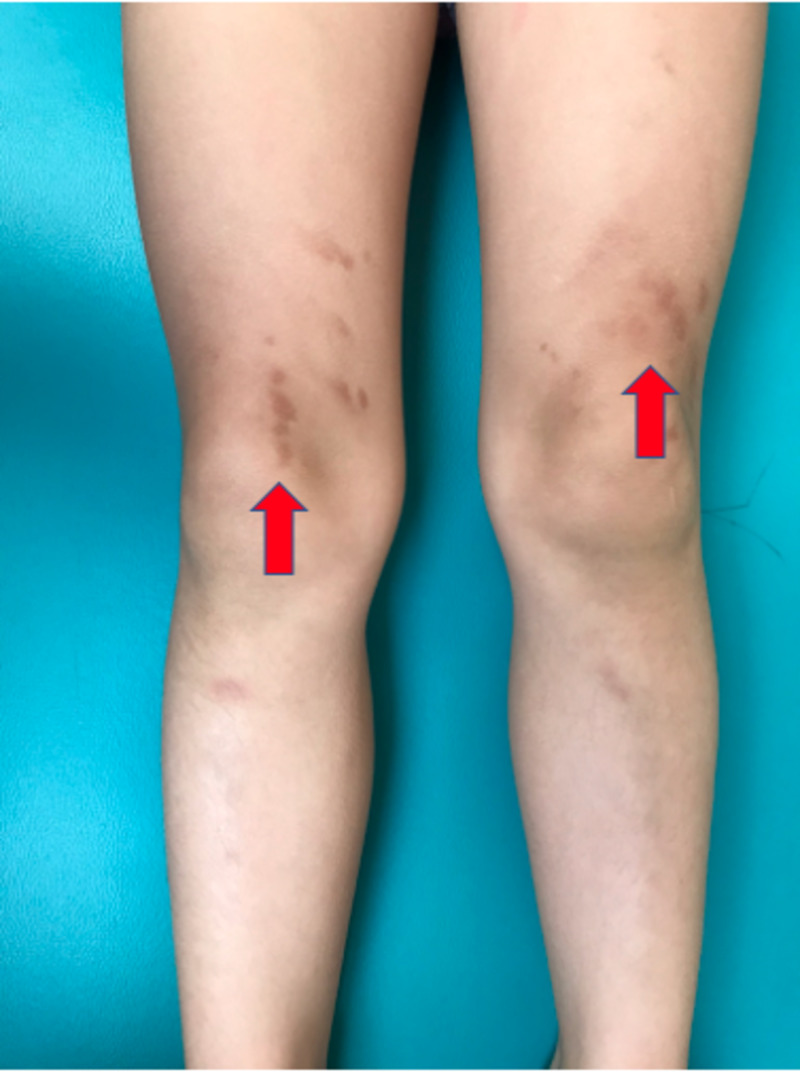
Hyperpigmented salmon-colored macules on the superior aspect of the knees bilaterally

An otherwise healthy, four-year-old, Hispanic female presented to the dermatology clinic with a one-week history of a pink-to-brown pruritic rash that presented on the hands and thighs after playing with Play-Doh and slime a few days prior. The patient's mother denied any pain, burning, or stinging sensation associated with the rash and reported no other significant exposure to fragrances or environmental contaminants that may cause similar cutaneous manifestations. Due to the lack of exposure to alternative irritative substances, and the subjective significant exposure to both Play-Doh and slime prior to the onset of the rash, these products, in particular, were deemed to be causative. Physical examination revealed hyperpigmented macules on the dorsum of the left hand, interphalangeal joints of the right second to fourth digits (Figures 1, 2), and bilateral thighs (Figure 3).

There were few pink macules and patches on the left thigh and just below the right knee. While the patch test is considered the gold standard for diagnosis of ACD, one was not performed in this instance as treatment with triamcinolone acetonide 1% cream twice daily was started empirically due to a high clinical suspicion, with significant symptomatic improvement. It should be noted that the absence of patch testing or skin prick testing is a limitation of the study; however, a follow-up one month later revealed complete resolution of the rash on the thighs with some residual hyperpigmented macules on the dorsum of both hands. While we acknowledge the limitations of the study due to the lack of confirmatory diagnostic testing, we believe this case can be used to illustrate the increasing incidence of new contact dermatitis cases secondary to both slime and Play-Doh, and help educate clinicians about proper management of this condition.

## Discussion

Contact allergic reactions are often eczematous but test positive on an immediate protein patch test. The most common allergens are nickel, fragrances, and preservatives [1,2]. Sensitization occurs via specialized dendritic cells known as LCs that react along with adjacent keratinocytes to allergens or haptens (large proteins that require a carrier molecule to elicit a response). Once these LCs recognize a specific allergen they prime naïve T cells, leading them to differentiate into allergen-specific CD8+ T cells, better known as cytotoxic T cells (CTLs). These CTLs will recognize this allergen for life and will react upon subsequent exposure by killing the allergen and keratinocytes. This hypersensitive immune response is mediated through the release of inflammatory cytokines IFN-y, and TNF-a. The role of CD4^+^ T-cells and regulatory T-cells is not clearly understood at this time, but they are believed to act cooperatively with the activated CD8^+ ^T-cells through activation of toll-like receptors (TLRs), namely hTLR4 [1]. Clinically, ACD can present as an acute or chronic reaction. Beginning 12 hours after exposure, an edematous pruritic skin rash develops, which can be accompanied by vesicles that crust over. In the setting of chronic, low-level exposure to the offending agent, ACD is characterized by itching, scaling, and lichenification [3]. In comparison with ACD, ICD is a localized inflammatory reaction that is triggered by repetitive encounters with irritating substances such as soaps and detergents. This leads to a non-immunologically mediated reaction, with subsequent manifestations ranging from local swelling to severe pruritis and erythema based on the strength of the irritant. Individuals with a background of atopy, particularly atopic dermatitis, are more susceptible to ICD as a result of the impaired barrier function of the skin [4]. In contrast to ACD, ICD does not require prior sensitization, as cutaneous reactions can occur immediately.

These very same mechanisms cause ACD due to wheat and flour additives, often seen in the pediatric and occupational setting. Flour contains a variety of immunogenic proteins, including alpha-amylase, beta-amylase, and amyloglucosidases among others [5]. Of these proteins, alpha-amylase has been well established to cause asthma, rhinitis, and dermal reactions. Concerning our case, alpha-amylase is a natural component of flour, which according to Hasbro Inc. is one of the main components of Play-Doh [6]. Children with wheat or flour allergies may develop ACD from playing with or ingesting Play-Doh.

“Slime” is another popular childhood toy often associated with contact dermatitis reactions. Similar to Silly Putty or Play-Doh, it is a gooey, gelatinous material popular among preteens and adolescents that is noted for its playful ability to stretch and morph in a variety of ways. While the main ingredients of slime vary as it can easily be made from home, the most common variations include glue (polyvinyl acetate) and borax (sodium tetraborate). Borax has been known to cause allergic reactions when used as an alternative laundry detergent; more recently, it has been demonstrated that borax may precipitate ICD rather than ACD, the differences which we discussed previously [7]. Glue ingredients are typically not available as most are proprietary formulations. However, one study recently found a compound, methylchloroisothiazolinone/methylisothiazolinone (MCI/MI), to be a common inclusion. These compounds are preservatives commonly found in personal care products, such as cosmetics, shampoos, and conditioners, along with most popular school glues. These have been found to be the causes in many cases of slime-related contact dermatitis. 

While over 100 years old, the patch test is still considered the gold standard for diagnosis. The skin prick test is an alternative method for diagnosing ACD, which is also widely used [8]. Biopsy of these lesions is rarely performed due to the sensitivity of the patch test and similar treatment modalities between the common differential diagnoses. However, a biopsy is indicated for cases that remain indeterminate after patch testing. It is important to differentiate the histopathology between ACD and ICD. The presence of focally distributed parakeratosis and bacteria in the corneal layer distinguishes ACD from ICD. In addition, deep dermal infiltrates and a lack of edema in the papillary dermis is more frequently encountered in ACD than ICD [9].

The mainstay of therapy of contact dermatitis caused by slime or Play-Doh is avoidance of these substances. The addition of oral antihistamines (cetirizine and loratadine typically) and topical corticosteroid creams (triamcinolone and mometasone) is often employed to relieve persistent cases [10,11]. The usage of topical corticosteroids should be restricted to the smallest amount necessary to resolve the rash, especially in children. In slow-resolving cases, topical corticosteroids may be applied up to four times per day. While absorption rates for topical corticosteroids, specifically triamcinolone, generally reside around 10%, long-term use should be avoided due to a slew of adverse effects that can develop with prolonged exposure [12]. 

## Conclusions

ACD is a cutaneous reaction to an allergen that is mediated via release of inflammatory cytokines TNF-a and IFN-y, after a preceding sensitization period mediated by LCs in the dermis. Systemic manifestations of the condition typically stem from an overproduction of these cytokines due to widespread activation of pre-sensitized or primed CD8^+^, CD4^+^, and regulatory T-cells producing keratinocyte apoptosis via the Fas/FasL cascade. Typically ACD is most often seen after exposure to nickel, fragrances, and preservatives, but it can arise after contact with childhood toys such as Play-Doh and slime, due to the variable nature of their composition. The gold standard of diagnosis for ACD is the patch test, as it is both non-invasive and cost-effective. Treatment of ACD is typically avoidance of the allergen substance. However, in patients with a diffuse rash (as seen in our patient), or when avoidance of the allergen is not possible, treatment should include topical corticosteroids. Our patient's lesions resolved with the use of triamcinolone acetonide 1%, which speaks to the efficacy of the treatment. The low potency of this corticosteroid combined with its cost-effectiveness further underscores its use as a first-line treatment option. We hope this case proves as a useful tool in helping physicians recognize ACD clinically, and helps to promote proper treatment regimens for adequate resolution of the patient's symptoms.
